# How to persuade more primary care professionals to adopt a valued smoking cessation referral aid: a cross-sectional study of facilitators and barriers

**DOI:** 10.1186/s12875-022-01843-3

**Published:** 2022-09-07

**Authors:** Daniëlle N Zijlstra, Catherine AW Bolman, Jean WM Muris, Hein de Vries

**Affiliations:** 1 Department of Health Promotion, Maastricht University/CAPHRI, Maastricht, Netherlands; 2 Department of Psychology, Open University of the Netherlands, Heerlen, Netherlands; 3 Department of General Practice, Maastricht University/CAPHRI, Maastricht, Netherlands

**Keywords:** Smoking cessation, Evidence-based interventions, General practice, Primary care, Nurse practitioners

## Abstract

**Background:**

To study the factors associated with the intention of primary care professionals (PCPs) to use or not use a referral aid (RA) for selecting an evidence-based smoking cessation intervention (EBSCI).

**Methods:**

Participants (*n* = 85) were recruited from June to September 2020 to complete an online questionnaire based on the I-Change Model to assess the factors associated with the adoption of RA. The differences between PCPs with (*n* = 37) and without (*n* = 48) the intention to adopt in terms of demographics, motivational factors, and post-motivational factors were subsequently assessed. Correlation and logistic regression analyses were conducted to investigate the factors associated with the intention to adopt.

**Results:**

Both groups indicated that they highly appreciated the RA. However, PCPs without the intention to adopt expressed a more negative attitude towards the RA, experienced less social support, showed low self-efficacy, and encountered barriers such as lack of time and skills. The factors most strongly associated with the intention to adopt were advantages, disadvantages, self-efficacy, less barriers, working in a solo practice and age.

**Conclusions:**

The adoption of RA can be facilitated in two ways. The first one is by increasing the added value of the tool through a second round of co-creation focusing on the adoptability of the RA in practice. The second approach is by communicating the added value of referring to EBSCIS and thereby using the RA by implementing it in smoking cessation training for PCPs, which could also help to improve the attitude, social support, self-efficacy, and perceived skills in terms of RA usage among PCPs.

**Impact:**

This study is the first work in the Netherlands to investigate the willingness of PCPs to actively refer patients to other EBSCIs in addition to providing face-to-face counseling themselves.

**Trial registration:**

The study was registered at the Netherlands Trial Register (NL7020, https://www.trialregister.nl/trial/7020).

## Background

Tobacco use continues to cause a range of noncommunicable diseases, and it is responsible for approximately eight million deaths worldwide every year [1]. The prevalence of daily smoking among adults in the Netherlands was approximately 18% in 2017 [1, 2]. The primary care setting (PCS) can play a significant role in smoking cessation, as most smokers visit their general practitioner (GP) yearly for a consultation with either the GP or a practice nurse (PN; [3]. PNs specialized in the treatment of chronic diseases are usually trained in providing smoking cessation counseling according to the Dutch Smoking Cessation Guidelines (DGSCC: [4, 5]; these guidelines are based on the internationally used protocol of 5As, namely Ask, Advise, Asses, Assist, and Arrange [6]. According to these guidelines, the PCS is also the main gateway for a prescription to pharmacotherapy (including nicotine replacement therapy (NRT)) and other evidence-based smoking cessation interventions (EBSCIs), some of which can also be administered outside of the PCS.

Although it is part of the smoking cessation guidelines, discussing EBSCIs within a consultation is still often skipped by both international [7, 8] and Dutch PNs [9]. This might be part of why only 19–25% of the Dutch smokers who are willing to quit make use of EBSCIs even though the use of EBSCIs significantly increases the success rate of smoking cessation attempts [4, 10]. Establishing clear guidelines on how to aid smoking patients in deciding the EBSCI that is the most appropriate for them may not only increase the referral to and use of EBSCIs and boost the patients’ commitment but may also facilitate a more complete implementation of smoking cessation guidelines in the PCS, reduce the time burden, and may lead to a more efficient process outside of practice in broader geographical areas. An approach based on informed and shared decision principles, rather than top–down recommendations or very brief advice by a primary care professional (PCP), may increase the referral to and use of EBSCIs. It may also enhance smoking patients’ involvement and commitment by providing them with the support that best fits their needs and preferences [11, 12]. Therefore, a referral aid (RA), the “*StopWijzer*” (which can be translated as stop-indicator and stop wisely in Dutch), was developed. The goal of the RA is to optimize the referral to and use of EBSCIs in the PCS and to increase the success rate of smoking cessation attempts. The aim of this study is to investigate the factors that influence the intention of PCPs to adopt a smoking cessation RA aimed at encouraging PCPs to refer patients to EBSCIs, both those offered inside and outside the PCS.

The RA consisted of an intervention manual with additional materials such as a flow-chart and a poster (described in further detail in the method section), which aims to guide PCPs in discussing smoking cessation with patients and aid them in selecting an EBSCI that fits the patients’ individual needs and preferences. The RA was based on a needs assessment comprising a literature review (e.g., [5, 9, 13, 14]; individual interviews with respectively GPs, PNs, and smokers; a Delphi study on the referral to EBSCIs in the PCS [15]; and input from the advisory board that was installed for the research project. The EBSCIs included in the RA, which are also referred to in the DGSCC [4, 5], are face-to-face counseling [16], online counseling (eHealth) [14, 17], telephone counseling [18], group counseling [19], pharmacotherapy [20–22], and NRT [23]. A brief chapter aimed to discourage the use of non-evidence-based interventions such as acupuncture and e-cigarettes to quit is also included. (For more information on the RA and the associated materials, see [24, 25].) The materials are designed to be used in addition to the support offered by the PCPs and not as a substitute for care-as-usual.The first step in the successful dissemination of interventions is successful adoption by the end users, in this case PCPs [26, 27]. Theories such as the theory on diffusion of innovations [28], the theory of planned behavior (TPB; [29], and the integrated change model (ICM) [13] are often used as theoretical frameworks to gain insight into the factors influencing the adoption of smoking cessation interventions among healthcare professionals (e.g., [30–35]. The diffusion theory of Rogers seeks to explain how an innovation spreads over time among potential adopters. The TPB predicts an individual’s intention to perform a certain behavior through motivational factors (attitude, subjective norms, and perceived behavioral control (PBC)). The ICM adds predisposing factors such as behavioral (past attempts of changing the behavior), psychological (personality traits), biological (age or gender), and environmental factors (policies). The ICM further adds (perceived) barriers, action or coping plans, and skills [13, 36–38].

Predisposing factors such as occupation [39], work experience, and time spent on counseling have been associated with adherence to smoking cessation guidelines [31]. In addition, Bolman and colleagues [32] have indicated that predisposing factors do not influence the intention to adopt directly when motivational factors are considered. Motivational factors entail attitude (i.e., advantages and disadvantages), self-efficacy, and social support or social norms. Earlier research revealed attitude to be a strong predictor for the intention to adopt [32–34, 39]. Self-efficacy has often been associated with the PCPs’ adoption of interventions to improve smoking cessation care. In some studies, higher self-efficacy has been associated with a higher adoption rate [30–35, 40]. However, other studies found no significant relationship between self-efficacy and adoption [39]. Social support or social norms are less often associated with the intention to adopt among GPs, although some studies found an association [39]. This association may be explained by the fact that PNs are frequently shown to be the only smoking cessation counseling point of contact within their individual PCS [31], as studies in other health care settings sometimes reported an association [34, 41]. Aside from motivational factors, perceiving fewer barriers in adopting a new smoking cessation RA [34] and being comfortable executing the steps that are part of an intervention [39] are found to be related to a higher intention to adopt. Other factors found to be associated with the intention to adopt a smoking cessation counseling aid include the belief that adoption is futile, as most smokers are unwilling to quit [42].

**Fig. 1:**
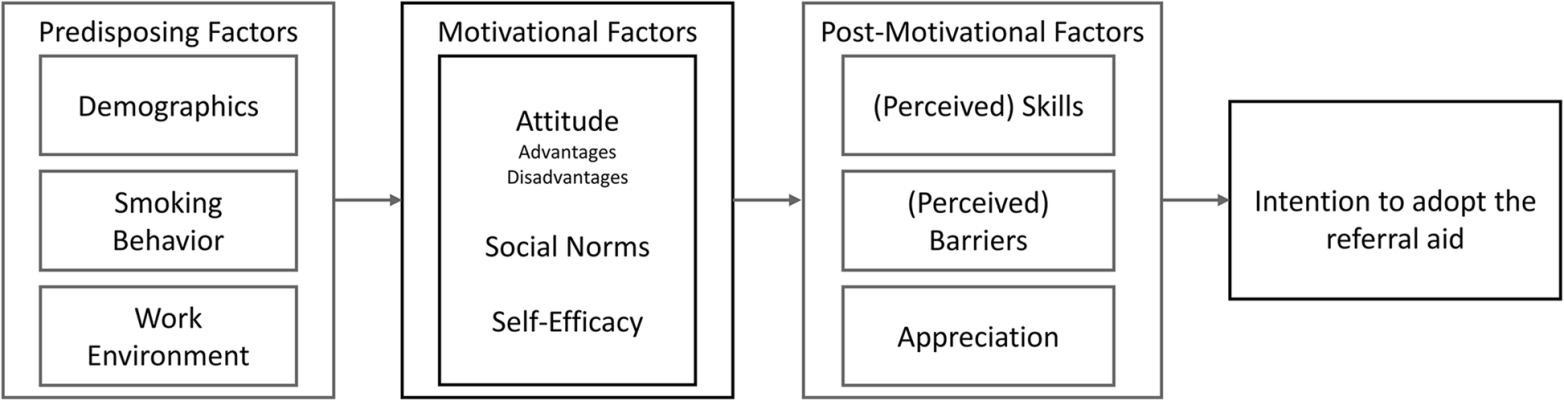
The ICM applied to the adoption of the RA by PCPs in the PCS

In contrast to the interventions in the aforementioned studies, this intervention focuses not only on smoking cessation counseling administered inside the PCS but also on the willingness of PCPs to actively refer patients outside of the PCS, as this aspect is a crucial innovation of the new RA. In this study, the ICM was used as a theoretical framework to assess the determinants of the intention to adopt (see Fig. 1).

## Methods

### Aims

The aim of this study was to investigate the factors that influence the intention of PCPs to adopt a smoking cessation intervention referral aid aimed at encouraging PCPs to refer patients to EBSCIs, both those offered inside and outside the PCS. In this regard, we studied the relevant motivational factors related to the intention to adopt, such as experiencing relatively more benefits (e.g., RA is an effective method of bringing EBSCIs to the smokers’ attention) and fewer disadvantages (e.g., the belief that the RA would not improve the quality of smoking cessation counseling), higher self-efficacy, and possibly higher social norms. In addition, we explored the PCPs’ degree of appreciation for the RA, the perception of PCPs’ skills in following the intervention steps, and the PCPs’ perceptions of barriers to referring patients to EBSCIs. As some studies indicated some influence of the predisposing factors, we included the factors that measure the PCPs’ own smoking behavior and their work environment as well as standard demographic measurements.

### Design

A cross-sectional survey was used in this study.

### Sample/participants

Two groups were invited via email, namely all the PCPs who were assigned to the control group in our randomized controlled trial and had therefore not yet worked with the RA (*n* = 32) [25] and the PCPs who were not involved in our RCT (*n* = 200). The additional 200 PCPs were recruited throughout the Netherlands using existing mailing lists. The participants received a link to an online questionnaire, a summary of the RAs’ content with screen shots of the associated materials, and a link to the RA website where all materials could be viewed. PCPs did not extensively use the RA before completing this study, but they were invited to use the materials in daily practice after the study if they desired to do so.

### Referral aid

The RA consisted of a manual that summarizes EBSCIs (i.e., counseling via GP, PN, or coach, eHealth, counseling via telephone, group counseling, NRT, pharmacotherapy) and non-evidence-based methods such as acupuncture and e-cigarettes to quit, their disadvantages, costs, and a list of PCPs within the Netherlands. The manual also encapsulates the steps recommended during a counseling session according to the DGSCC [4, 5], the Dutch healthcare reimbursement system, and some recommendations for planning and conducting a follow-up meeting. Additional materials for the PCP comprised a laminated option grid (designed to be used as a table card) in A3 format, a summarization of the RA protocol in A5 format, and various promotional materials such as a (digital) poster in the practice waiting room, flyers, and business cards (not specifically evaluated during this study).

The RA protocol included five steps (excluding the steps that were only undertaken as part of the data collection for the related RCT study [25]. First, the participating PCPs identified patients with smoking-related complaints and asked whether they were motivated to quit smoking. Second, when smoking patients were willing to talk about smoking cessation, PCPs were tasked with explaining the EBSCIs in accordance with the RA materials. Third, when relevant, PCPs were stimulated to explain the Dutch reimbursement system for smoking cessation methods outside of the PCS. Fourth, when smoking patients expressed a preference for one of the recommended EBSCIs, PCPs referred them to the appropriate interventions as described in the RA. Finally, PCPs were advised to schedule a follow-up meeting a few weeks after the decision to talk about the patient’s experiences and progress.

### Data collection

Data were collected from June until September 2020 via an online questionnaire that could be accessed through an online platform (Formdesk, www.formdesk.nlhttp://www.formdesk.com/). Informed consent for participation and the use of data was obtained by affirmatively answering one question to access the questionnaire; PCPs who did not give consent were excluded from the study. The questionnaire took on average 15 min to complete, excluding the time that PCPs needed to explore the materials. PCPs who did not respond within seven days were sent a maximum of two reminders. Upon the completion of the questionnaire, participants received a reimbursement of €20 in gift vouchers.

### Questionnaire

The questionnaire consisted of 41 questions. These questions concerned PCPs’ demographic characteristics (including their own smoking behavior and work environment), intentional factors, motivational factors, and factors regarding perceived skills, perceived barriers, and appreciation (post-motivational factors, explained in more detail below).

#### Demographics and smoking characteristics

In terms of demographic variables, PCPs were queried about their gender, age, occupation (e.g., practice nurse/nurse specialist, or other) and the number of years they had been active in that occupation. The PCPs’ own smoking behavior was assessed using one item (smoker/ex-smoker/non-smoker). The PCPs’ work environment (e.g., type of PCS, such as a solo practice; refer to Table 1 for a complete overview) was assessed using six items.

**Table 1:** Comparison of demographics and smoking characteristics between adopters and non-adopters

	**Total (*n* = 85)**	**Adopters (*n* = 37)**	**Non-Adopters (*n* = 48)**	**T-test**	**χ2**	***P***
**Demographics**						
Gender female (%)	84 (98.8)	36 (97.3)	48 (100)			.435^a^
Mean age in years (SD)	45.5 (11.6)	48.3 (11.1)	43.2 (11.7)	-2.041		.044
Occupation (%)						.521^a^
• PN/Nurse specialist	74 (87.1)	31 (83.8)	43 (89.6)			
• Other^b^	11 (12.9)	6 (16.2)	5 (10.4)			
Mean years active in occupation (SD)	10.05 (6.4)	11.7 (7.3)	8.8 (5.4)	-2.120		.037
Smoking behavior (%)						
• smoker	3 (3.5)	1 (2.7)	2 (4.2)		-	-
• ex-smoker	27 (31.8)	17 (45.9)	10 (20.8)		6.079	.014
• non-smoker	55 (64.7)	19 (51.4)	36 (75.0)		5.117	.024
**Work environment**						
Type of general practice (%)					3.576	.167
• Solo practice	20 (23.5)	12 (32.4)	8 (16.7)			
• Group practice	40 (47.1)	17 (45.9)	23 (47.9)			
• Other^c^	25 (29.4)	8 (21.6)	17 (35.4)			
Mean years active in practice (SD)	9.68 (7.8)	10.8 (6.8)	8.8 (8.4)	-1.179		.242
Mean working hours per week (SD)	25.40 (7.1)	27.4 (6.7)	23.8 (7.1)	-2.370		.020
Mean number of patients in practice (SD)	5382.54 (2944.1)	5250.0 (3196.8)	5491.5 (2751.1)	0.368		.714
Mean number of smokers per practice who in general receive brief/short smoking cessation advice per month (SD)	10.94 (8.4)	11.6 (10.1)	10.4 (6.8)	-0.667		.507
Mean number of smokers per practice who receive smoking cessation counseling per month (SD)^d^	4.15 (3.7)	4.9 (5.1)	3.6 (2.1)	-1.619		.109

^a^ Fisher’s Exact Test reported as numbers are insufficiently high for calculating χ^2^

^b^ e.g., Practice assistant, General practitioner or other (individual groups too small to perform separate analyses)

^c^ e.g., Health center, medical center, general practitioner with dispensing pharmacist

^d^ This means that we asked PN to estimate the absolute number of active counseling by the PCP according to the Dutch Guidelines for smoking cessation; this might also include the prescription of pharmacotherapy

#### Intentional factors

To compare participants based on their intention to adopt the RA, intention to adopt was assessed using three items (e.g., “I consider it likely that the RA will be implemented in practice”). All intention items were measured using a five-point Likert scale ranging from 1 (“surely not”) to 5 (“most certainly yes”). Based on the three items, a mean score was formed (Cronbach’s α = 0.78).

#### Motivational factors

The advantages of the RA (e.g., “The RA is easy to apply in daily practice”) were measured with four items using a five-point Likert scale ranging from -2 (“completely disagree”) to 2 (“completely agree”). Moreover, the items were combined into an overall advantages scale using the mean score (α = 0.73).

The disadvantages of the RA (e.g., “The RA does not improve the quality of providing smoking cessation information in general practice”) were measured with four items using a five-point Likert scale ranging from -2 (“completely disagree) to 2 (“completely agree”). Additionally, the items were combined into an overall disadvantages scale using the mean score (α = 0.80).

Social support towards using the RA in daily practice from GPs, PNs, assistants, and the care group in which the participant worked was assessed using four items (e.g., “When implementing the RA in practice, I expect much opposition or support from my (fellow) PNs”). All social support items were measured using a five-point Likert scale ranging from 1 (“much opposition”) to 5 (“much support”) and combined into an overall scale using the mean score (α = 0.74).

Self-efficacy towards using the RA in daily practice was assessed using four items (e.g., “I find difficulty in using the RA when the patient is clearly not motivated to stop smoking”). All self-efficacy items were measured using a five-point Likert scale ranging from 1 (“very difficult”) to 5 (“very easy”). The reliability analysis showed a low reliability (α = 0.52); however, given the exploratory nature of this study, the scale was used in the regression analysis.

#### Post-motivational factors

Perceived skills necessary to successfully implement the steps as shown by the RA protocol were assessed using five items (e.g., “I think I am able to identify a patient as a smoker who is motivated to quit smoking”) using a five-point Likert scale ranging from 1 (“very difficult”) to 5 (“very easy”). Moreover, the items were merged into a mean skills scale (α = 0.65).

Perceived barriers towards adopting the RA in daily practice were assessed using five items (e.g., “There is too little time to use the referral guide”). All the barriers’ items were measured using a five-point Likert scale ranging from 1 (“totally disagree”) to 5 (“totally agree”) and were merged by forming a scale using mean scores (α = 0.71) after the exclusion of one item (i.e., “Many patients in our practice have an insufficient command of the Dutch language”). The language item was included as a separate item.

Appreciation was assessed using four items, of which three were measured (i.e., “I found the RA materials clear/understandable/instructive”) using a five-point Likert scale (1 = totally disagree; 5 = totally agree). The three items were combined into a mean appreciation scale (α = 0.86). Participants were additionally asked to rate the intervention materials in total on a scale of 1 to 10 (1 = bad; 10 = very good).

### Data analysis

Descriptive statistics were used for describing the characteristics of the participants. Participants were divided into two groups, PCPs with (certainly and most certainly) and without (surely not, not, and neutral) the intention to adopt the RA. T-tests and chi-square tests were conducted to assess the potential differences between both groups in terms of demographics, motivational factors, and post-motivational factors. To control for multiple testing, we used a significance criterion of *P* < 0.01.

Next, correlations between all relevant concepts (predisposing, motivational and post motivational variables) and the intention to adopt were tested via Pearson correlation coefficient. Variables that revealed significant correlations with the outcome measure (age, years active in occupation, being an ex- or non-smoker, working in a solo practice or other type of practice (non-group), years active in practice, advantages and disadvantages, social support, and self-efficacy, perceived skills and barriers) were used in a linear regression analysis using forward stepwise selection to determine predictors of intention to adopt the RA.

### Validity, reliability and rigour

Questions on motivational factors were based on relevant existing scales [34, 41, 43]. Questions to measure the appreciation of the RA were also used in related studies among smoking patients [24, 25] to facilitate the comparison.

## Results

### Demographics and smoking characteristics

The recruitment process resulted in 85 participants (response rate = 34%), from whom *n* = 18 stemmed from the original control group (response rate = 56%) and *n* = 67 from the newly approached group (response rate = 34%). The participating group of PCPs consisted of 4 GPs, 46 PNs, 3 practice assistants, 28 nurse specialists, and 4 others. The participants worked on average 25.4 h (SD = 7.1) per week in the PCS.

Participants were divided into PCPs with (*n* = 37, 43.5%) and without (*n* = 48, 56.5%) the intention to adopt. In the group of without intention, PCPs were significantly younger, worked for a shorted duration in their described occupation, worked fewer hours, and were less often ex-smokers (for all the study demographics, see Table 1).

### Differences between PCPs with and without the intention to adopt

#### Motivational factors

PCPs without intention perceived fewer advantages than PCPs with intention, as they less often reported that (1) the RA was an effective method of supporting patients in their choices about quitting smoking, (2) the RA was easy to apply in daily practice, and (3) the RA helped them to bring smoking cessation to their patients’ attention in a more effective manner. PCPs without intention also reported more disadvantages. First, they indicated that the RA was not sufficiently enabling patients to make an informed choice about quitting smoking. Second, they stated that the RA increased the risk that patients no longer want to quit smoking. Third, they believed that the RA was difficult to apply. Furthermore, PCPs without intention perceived less support from their environment, especially from other PCPs. Finally, PCPs without intention reported a lower self-efficacy, especially with regard to situations in which they are very busy or when the patient is not motivated to stop smoking. An overview of all the differences is presented in Table 2.

**Table 2:** Significant differences in mean scores between adopters and non-adopters on motivational factors (attitude, social support and self-efficacy)

	**Overall Mean (SD)**	**Adopters Mean (SD)**	**Non-adopters Mean (SD)**	**T-test**	***P* value**
**Advantages**^a^	0.79 (0.5)	0.84 (0.5)	0.73 (0.4)	3.455	.001
The referral aid:					
• is a good method to support patients in their choices about EBSCIs	0.99 (0.6)	1.05 (0.7)	0.91 (0.5)	2.092	.004
• is easy to apply in daily practice	0.93 (0.6)	0.93 (0.6)	0.91 (0.6)	2.680	.009
• helps me to better bring smoking cessation to the attention of smoking patients	0.30 (0.7)	0.41 (0.7)	0.15 (0.7)	2.993	.004
• helps patients to make a good choice about how they want to quit smoking	0.95 (0.6)	0.96 (0.6)	0.95 (0.5)	1.982	.051
**Disadvantages** ^a^	-0.55 (0.6)	-0.57 (0.7)	-0.53 (0.5)	3.556	.001
The referral aid:					
• does not improve the quality of smoking cessation information	-0.22 (0.9)	-0.30 (0.9)	-0.16 (1.0)	0.485	.006
• Does not help patients to make a good choice about quitting smoking	-0.39 (0.8)	-0.46 (0.8)	-0.34 (0.8)	3.552	.001
• Increase the risk that the patient will no longer wants to quit smoking with my help	-0.68 (0.8)	-0.70 (0.9)	-0.67 (0.8)	2.577	.012
• Is difficult to apply	-0.90 (0.8)	-0.95 (1.0)	-0.83 (0.6)	2.559	.012
**Social support**^b^	3.97 (0.7)	4.27 (0.7)	3.7 (0.6)	3.809	.000
• From the GP (or fellow GPs)	3.78 (0.9)	4.14 (0.9)	3.5 (0.8)	3.589	.001
• From the PN (or fellow PNs)	4.26 (0.9)	4.65 (0.8)	4.0 (0.9)	3.701	.000
• From the practice assistant (or fellow practice assistants)	3.94 (0.9)	4.14 (0.9)	3.8 (0.9)	1.707	.092
• From the care group to which the practice is affiliated	3.91 (0.9)	4.16 (0.9)	3.7 (0.9)	2.244	.028
**Self-efficacy**^c^	3.07 (0.5)	3.24 (0.5)	2.87 (0.5)	3.33	.001
I think it is more difficult to counseling smoking patients…					
• If I am very busy	3.24 (0.8)	3.49 (0.7)	3.04 (0.8)	2.757	.007
• If the patient is not motivated to stop smoking	2.82 (0.8)	3.05 (0.9)	2.65 (0.8)	2.254	.027
• If the patient is poorly educated	2.92 (0.9)	3.11 (0.9)	2.77 (0.9)	1.728	.088
• If I think that this means that you can no longer guide the patient yourself in quitting smoking?	3.14 (0.8)	3.30 (0.7)	3.02 (0.8)	1.685	.096

^a^ -2 = completely disagree; 2 = completely agree

^b^ 1 = much discouragement; 5 = much support

^c^ 1 = very difficult; 5 = very easy

#### Post-motivational factors and appreciation

PCPs without intention significantly differed from PCPs with intentionin their rating on the perceived skills scale and their rating on Step 2. Furthermore, PCPs without intention perceived significantly more barriers than PCPs with intention, except for patients with an insufficient command of the Dutch language, which did not significantly differ between both groups.

With regard to the total scale of appreciation, no significant differences were found between the appreciation of the RA by both groups (see Table 3). PCPs without intention gave the RA a slightly lower overall evaluation (8.04 out of 10) than PCPs with intention (8.69 out of 10) (T = 7.13, *P* < 0.05).

**Table 3:** Significant differences in mean scores between adopters and non-adopters on perceived skills, barriers, and appreciation

	**Overall Mean (SD)**	**Adopters Mean (SD)**	**Non-adopters Mean (SD)**	**T-test**	***P***
**Perceived skills**^a^	3.87 (0.4)	4.03 (0.4)	3.74 (0.3)	3.524	.001
• **Step 1*:*** Identify a patient as a smoker who is motivated to quit smoking	3.88 (0.6)	4.00 (0.6)	3.79 (0.6)	1.586	.117
• **Step 2:** Explain smoking cessation methods using the referral aid	3.92 (0.5)	4.08 (0.5)	3.79 (0.5)	2.651	.010
• **Step 3:** Provide an explanation of the reimbursement for external EBSCIs	3.69 (0.7)	3.89 (0.7)	3.54 (0.7)	2.264	.026
• **Step 4:** Refer the patient to an appropriate EBSCI with the help of the referral aid	3.81 (0.6)	4.00 (0.7)	3.67 (0.6)	2.429	.017
• **Step 5:** Contact the patient again after a few weeks to follow-up	4.02 (0.6)	4.16 (0.5)	3.92 (0.6)	2.057	.043
**Perceived barriers (scale)**^b^	2.22 (0.5)	1.94 (0.5)	2.44 (0.5)	-4.898	.000
• In our practice there are not enough smoking patients to use the referral aid meaningfully	2.00 (0.7)	1.73 (0.5)	2.21 (0.7)	-3.567	.001
• In our practice there is too little time to use the referral aid on smoking patients	2.28 (0.8)	2.03 (0.7)	2.48 (0.8)	-2.808	.006
• We do not have enough staff in our practice to use the referral aid consistently	2.28 (0.7)	2.00 (0.7)	2.50 (0.7)	-3.378	.001
• Patients in our practice have little interest in discussing smoking cessation possibilities	2.33 (0.8)	2.00 (0.5)	2.58 (0.8)	-3.676	.000
**Perceived barriers (other)**					
• Many patients in our practice have insufficient command of the Dutch language	1.98 (0.9)	2.03 (0.9)	1.94 (0.8)	0.474	.637
**Appreciation**^c^	3.85 (0.6)	3.97 (0.7)	3.75 (0.4)	1.854	.067
• I think the referral aid materials are clear	3.93 (0.6)	4.05 (0.7)	3.83 (0.6)	1.663	.100
• I think the referral aid materials are understandable	3.93 (0.6)	4.05 (0.7)	3.83 (0.5)	1.720	.089
• I think the referral aid materials are educational	3.68 (0.7)	3.81 (0.8)	3.58 (0.6)	1.550	.125
• I grade the referral aid materials with a mark of (0–10)	8.48 (1.1)	8.78 (1.3)	8.25 (0.8)	2.302	.024

^a^ 1 = Very difficult; 5 = very easy

^b^ 1 = totally disagree; 5 = totally agree

^c^ 1 = totally disagree; 5 = totally agree

### Factors (uniquely) associated with the intention to adopt

Intention to adopt was most strongly positively correlated with perceived advantages (*r* = 0.53), social self-efficacy (*r* = 0.49), support (*r* = 0.47), skills (*r* = 0.33), being an ex-smoker (*r* = 0.31), years active in occupation (*r* = 0.30), working in a solo-practice (*r* = 0.21), years active in practice (*r* = 0.19) and age (*r* = 0.19). Perceived disadvantages (*r* = -0.54), the perception of (many) barriers (*r* = -0.50), being a non-smoker (*r* = -0.32) and working in another type of practice (not solo or group) (*r* = -23) had a negative correlation with the intention to adopt. The results of a forward linear regression revealed that factors most strongly associated with the intention to adopt were advantages (β = 0.39), disadvantages (β = -0.36), self-efficacy (β = 0.35), less barriers (β = -0.27), working in a solo practice (β = -0.23) and age (β = 0.01). The overall model explained 63% of the variance.

## Discussion

In this study, the factors influencing the PCPs’ intention to adopt a new smoking cessation RA were examined using the ICM as a theoretical framework. Although the appreciation in both groups was high (both groups scored the RA materials higher than an 8), most PCPs did not intend to adopt the RA (*n* = 48, 56.5%). The PCPs without intention in the sample reported an overall more negative attitude towards the RA (more disadvantages and fewer advantages) than PCPs with intention, experienced less social support and a lower self-efficacy, while also experiencing more barriers to adopt and having less perceived skills, factors that all indicated to correlate with the dependent variable of intention.

A positive attitude (perceiving more advantages and fewer disadvantages) towards an innovation is a well-documented factor of adoption both among PNs [39, 44] and other general practice staff [33, 41] or health professionals outside of the general practice setting [32, 34]. In our sample, PCPs without intention were least convinced that the RA would help them to bring the topic of EBSCI usage to the attention of the smoking patient. This result might be because raising such topic is not part of their current routine or because they feel they have already inquired about the smoking status of most of their patients and therefore are unwilling to discuss this subject any further [42]. We were unable to confirm this finding based on our outcomes. The most important disadvantage considered in both groups was the perception that the RA would not improve the quality of smoking cessation information or counseling in general practice. PCPs without intention were even significantly more convinced of this idea. As PCPs and especially PNs are expected to provide counseling in accordance with the DGSCC [4, 5], in which referral is already one of the steps, perhaps the PCPs do not see the added value of the RA in their counseling, for example because they do not recognize the added value of EBSCIs themselves, or they are already familiar with the different EBSCI options and thus are substantially knowledgeable about what they can denote. The DGSCC [4, 5] are designed as an overall guideline, and they do not provide specifics on the availability and costs of EBSCIs. Although they do include information on the effectiveness in the form of evidence tables, these tables might be difficult to understand and use in conversation. As the RA was developed as a facilitating tool for communicating this information (e.g., effectiveness and availability, among others) to smoking patients and rated as such in this study (perceived skills: Step 2), we assume that the RA can be of assistance in their daily routine to discuss EBSCIs with smoking patients. PCPs might have been insufficiently aware of this specific value of the RA. Aside from a positive attitude, a stronger intention to change is, according to the ICM [13], characterized by high levels of social support and self-efficacy.

We found that social support influenced the intention to adopt, especially support from the GP or PNs. Findings on the role of social support are equivocal in other studies, as some studies identify an influence [41], whereas others do not, possibly because of mediation by other factors [32–34, 39]. An explanation for the inconsistent findings may be the highly independent working environment of Dutch PCPs and PNs. In the Netherlands, approximately 88% of the PCS employ one or more PNs specialized in smoking cessation (the numbers are lower for solo practices) who work an average of three days a week [45]. As this case indicates that often only one PN is working per practice, regular communication with peers may be hindered. However, this research did not investigate the form that this support should assume.

Empirical evidence is inconsistent regarding the influence of self-efficacy on adoption, as some studies report no effect of self-efficacy on adoption [32, 34], whereas others have found an effect of high self-efficacy on adoption rate [30, 31, 33, 35, 46, 47]. In our sample, PCPs without intention reported the lowest self-efficacy to counsel smoking patients using the RA in case they were very busy, which was also reported in earlier research in a similar sample [42]. As PCPs get only a limited amount of time for counseling, especially for “healthy” smokers (smokers without smoking-related illnesses), a means of increasing the PCPs’ self-efficacy is by making sure that the smoking cessation counseling protocol is as easy and efficient as possible, for example by using the Ask-Advise-Refer strategy [48], in which the time-consuming counseling is conducted by another health care professional. This strategy has been proven effective in Dutch cardiac wards [34]. Increasing the timeframe that PCPs can spend per patient under full reimbursement may also help PCPs to overcome the time problem. However, our self-efficacy items turned out to have a low-test score reliability according to Cronbach’s alpha.

A regression analysis exploring factors explaining the intention to adopt revealed that intention to adopt is explained by perceiving more advantages, fewer disadvantages, more self-efficacy, less barriers, more often working in a solo practice and, at only a small rate, a higher age. As also found in previous research, the influence of the predisposing factors probably did not influence the intention to adopt directly when motivational factors were considered [32]. The overall model ultimately explained 63% of the variation in the intention to adopt between PCPs with and without intention, which is in line with comparable studies in other target groups (i.e. cardiac nurses, midwives [34, 41].

Yet, these results also reveal that a significant proportion of the variance is left unexplained, indicating that other factors are relevant as well, such as, occupation (could not be explored in our study because of a low amount of GPs in our sample) or time spent working after following a smoking-cessation counseling training (possibly related to age and time spent working in occupation), which have been found to be associated with adoption in previous studies [30, 39]. It is suggested to identify them, e.g., by conducting qualitative research, as well as performing a longitudinal study to identify predictor of adoption.

### Limitations

The added value of this study is the investigation of factors which influence the PCPs intention to adopt a smoking cessation RA aimed at stimulating PCPs to refer patients to EBSCIs, both those given within the PCS as outside of the PCS. However, this study had some limitations. First, as we used a cross-sectional design (one point in time, one measurement per respondent), we cannot draw causal conclusions. Secondly, we primarily used quantitative research methods to determine relevant factors related to the adoption of the RA, therefore only including factors that are present in the I-Change model. Using a mixed method study by including also qualitative research methods such as interviews might have provided us more factors that would be of influence for intention to adopt the RA. However, this might have resulted into a lower response rate by the increased burden on the participants. It is worthwhile mentioning that we actually based our selection of factors on semi-structured interviews with GPs (*n* = 5), PNs (*n* = 20), a Delphi study on the EBSCIs referral [15] and the input of our advisory board of Dutch experts from various smoking cessation related organizations. This provided some inside in the relevance of the factors included. Thirdly, we experienced a low response rate that resulted in a low sample size for the complete model testing. However, due to the explorative nature of this study and the strong evidence of using the I-Change model as a basis for explaining the intention to adoption as also seen in other studies [30–35], we decided to test the full model. Second, the sample of potentially more motivated participants selected from the population of Dutch PCPs who were willing to participate in this study and are expected to have seen the RA materials may constitute a limitation. Therefore, the results may not be generalizable to PNs and PCS in general. This is also reinforced by the small number of GPs, which prevented us from exploring the differences between different groups of professionals, thereby also limiting the homogeneity of our sample. Furthermore, as only Dutch PCPs were included in the sample, the results may only be relevant in the Netherlands. Finally, as this study used self-reported data, social desirability might have influenced the PCPs’ answers, for example resulting in a higher intention to adopt.

### Recommendations for future adoption

Although there was a large percentage of PCPs without intention to adopt the RA is the choice for the use of an RA still well substantiated based on theory related to (inter)national research on smoking cessation guidelines [4, 5], shared decision making principles [11, 12] and experiences from experts in the field [15]. As the appreciation for the RA was also high in both groups, we do see it feasible to continue in the proposed direction. To make the RA eligible for widespread adoption, it needs to be enclosed with motivational-enhancing communication to establish a more positive attitude towards the tool. A more positive attitude can be achieved by emphasizing the benefits of the referral tool (e.g., convincing the PCPs of the usefulness of the referral tool in helping smoking patients to choose an EBSCI) when introducing the referral tool to potential users to convince potential adopters of the RA’s added value. The prerequisite is that both the RA has added value and that the PCP and the patients alike recognize the added value of the RA compared to usual care. The proven effectiveness of the RA seems to be necessary in this case to facilitate its adoption and implementation. A means of increasing the added value of the RA is, for example, conducting additional research in the form of co-creation. Although the materials of the RA were created through co-creation sessions with PCPs, a second round of co-creation may be desirable. This round should then specifically focus on further developing the RA in a way that it can be more easily adopted into the PCS.

An approach to the facilitation of RA adoption is through the structural dissemination of the RA via existing sources such as relevant national training programs focused on smoking cessation counseling for the PCS, such as the one provided by the Dutch Quit Smoking Quality Register (kwaliteitsregister stoppen-met-roken, www.kwaliteitsregisterstopmetroken.nl). The quality registry lists qualified PCPs who are specially trained and experienced in providing intensive evidence-based counseling. To be listed in the registry, a training certificate must be obtained. Although the use of EBSCIs is already part of this training, the RA can help to increase (1) the awareness about EBSCIs, (2) the attitude towards referring to EBSCIs, (3) perceived social support among colleague PCPs also referring to EBSCIs according to the RA, and (4) self-efficacy by learning how to best implement the RA. The extent to which the RA already fits within this training or what is needed to implement it can be examined in the previously suggested second co-creation round.

Implementing the RA into the PCP quality training could also aid PCPs in discussing EBSCIs or smoking cessation in general with patients who are not motivated to quit, which is a known barrier in smoking cessation counseling (Blumenthal, 2007) and the most reported barrier in the current study. In the RA, we have endeavored to address the issue of motivating smokers who are unwilling to quit smoking by including one page of information describing the elements of motivational interviewing in our materials (e.g., how to motivate an unmotivated smoker to talk about smoking cessation), a technique that has proven to be effective when talking to non-motivated patients [49]. As motivational interviewing is also part of the PCP quality training, it could aid the PCPs’ technique in discussing the use of EBSCIs during a smoking cessation attempt. Improving the PCPs’ technique might also positively influence their perceived self-efficacy and skillset.

Another well-known and often reported barrier that was also noted in this study is a lack of time to provide more intensive counseling. This barrier fits well with the aim of the RA to reduce active counseling time by the PCP by referring patients to another specialist or EBSCI. Another reason for the low intention to adopt may also be that PCP's are reluctant to change part of their role to a more facilitative and referral role, possibly also because the Dutch financial reimbursement system does not reinforce this. Thus, more in-depth research on this topic may be needed, including a more in-depth qualitative approach targeting PCPs, but also health insurers in order to thoroughly assess the feasibility of the approach taken in our intervention. It would also be a good addition to investigate whether PNs actually intend to refer to EBSCIs and what the underlying reasons for this would be. Solutions to facilitate the RA usage include increasing the reimbursed time for smoking cessation counseling offered by PCPs, providing additional training to increase efficiency without compromising effectiveness [9, 35, 50, 51], and entrusting intensive counseling to specialized smoking cessation coaches with expertise in addiction care. Providing additional training could also help to strengthen the self-efficacy and perceived skills of PCPs without intention.

## Conclusion

This study examined the factors underlying the PCPs’ intention to adopt an RA by comparing PCPs with and without intention to adopt. Although appreciation in both groups was high (both groups scored the RA materials higher than an 8), most PCPs did not intend to adopt the RA. A regression analysis exploring the factors associated with the intention to adopt revealed that PCPs without intention perceived fewer advantages, showed lower self-efficacy, experienced less social support, and perceived more disadvantages. Recommendations for future adoption include improving the tool itself through a second round of co-creation focusing on the adoptability of the RA in practice. A second recommendation relates to communicating the added value of referring to EBSCIS and integrating the RA use in smoking cessation training for PCPs. This approach may help to improve the attitude, social support, self-efficacy, and perceived skills in terms of RA usage among PCPs.

## Data Availability

The datasets used and/or analysed during the current study are available from the corresponding author on reasonable request.

## Abbreviations

DGSCC: Dutch Smoking Cessation Guidelines
EBSCI: Evidence-based smoking cessation intervention
GP: General practitioner
NRT: Nicotine replacement therapy
PCP: Primary care professionals
PCS: Primary care setting
PN: Practice nurse
RA: Referral aid
TPB: Theory of planned behavior
ICM: Integrated change model
